# Breastfeeding mothers with COVID-19 infection: a case series

**DOI:** 10.1186/s13006-020-00314-8

**Published:** 2020-08-08

**Authors:** Augusto Pereira, Sara Cruz-Melguizo, Maria Adrien, Lucia Fuentes, Eugenia Marin, Azul Forti, Tirso Perez-Medina

**Affiliations:** 1 Department of Obstetrics and Gynecology, Puerta de Hierro University Hospital, Madrid, Spain; 2 Autonoma University of Madrid, C/ Manuel de Falla, 1, 28222 Majadahonda, Madrid, Spain; 3 Maternal-Fetal Medicine Unit, Department of Obstetrics and Gynecology, Puerta de Hierro University Hospital, Madrid, Spain; 4 Department of Psychiatry, Puerta de Hierro University Hospital, Madrid, Spain

**Keywords:** Breastfeeding, Case series, SARS-CoV 2, COVID-19, Breast milk, Donor breast milk, infant formula

## Abstract

**Background:**

The first reports of the Chinese experience in the management of newborns of mothers with SARS-CoV 2 infection did not recommend mother-baby contact or breastfeeding. At present, the most important International Societies, such as WHO and UNICEF, promote breastfeeding and mother-baby contact as long as adequate measures to control COVID-19 infection are followed. In cases where maternal general health conditions impede direct breastfeeding or in cases of separation between mother and baby, health organizations encourage and support expressing milk and safely providing it to the infants.

**Methods:**

A series of 22 case studies of newborns to mothers with COVID-19 infection from March 14th to April 14th, 2020 was conducted. Mothers and newborns were followed for a median period of 1.8 consecutive months.

**Results:**

Out of 22 mothers, 20 (90.9%) chose to breastfeed their babies during hospital admission. Timely initiation and skin to skin contact at delivery room was performed in 54.5 and 59.1%, respectively. Eighty two percent of newborns to mothers with COVID-19 were fed with breast milk after 1 month, decreasing to 77% at 1.8 months. Six of 22 (37.5%) mothers with COVID-19 required transitory complementary feeding until exclusive breastfeeding was achieved. During follow-up period, there were no major complications, and no neonates were infected during breastfeeding.

**Conclusions:**

Our experience shows that breastfeeding in newborns of mothers with COVID-19 is safe with the adequate infection control measures to avoid mother-baby contagion. Supplementing feeding with pasteurized donor human milk or infant formula may be effective, until exclusive breastfeeding is achieved.

## Background

Severe acute respiratory syndrome coronavirus 2 (SARS-CoV-2) is an RNA virus that causes coronavirus disease 2019 (COVID-19) [1]. The first COVID-19 case was reported on Dec 31, 2019 [2], and to date more than 11 million people worldwide have been affected by COVID-19 [3].

The World Health Organization (WHO) declares breastmilk as the ideal food for infants because is safe, clean and contains antibodies which help protect against many common childhood illnesses. Breastmilk provides all the energy and nutrients that the infant needs for the first months of life, and it continues to provide up to half or more of a child’s nutritional needs during the second half of the first year, and up to one third during the second year of life.

Since March 18th, 2020, the WHO recommends that women with COVID-19 can breastfeed if they wish to do so, based on the idea that through breastmilk the babies would get antibodies and anti-infective factors that help protect newborns from getting infections [4]. The WHO encourages women to breastfeed or to continue breastfeeding following certain recommendations and precautions [4].

Currently, it is uncertain whether the virus can be transmitted through breastmilk; our understanding of viral transmission is limited and based on a few reports where they did not find traces of the virus in breastmilk [5–7].

Also, there is some disagreement in the literature among breastfeeding management in confirmed COVID-19 patients, Wang et al., did not recommend breastfeeding in suspected cases, uncured cases and while taking lopinavir/ritonavir treatment [7]. In addition, neonatal isolation was recommended in newborns who had been suspected or confirmed SARS-CoV 2 infection [8]. On the other hand, the Union of European Neonatal and Perinatal Societies advise direct breastfeeding under strict measures of infection control in asymptomatic COVID-19 mothers, but when the mothers are too sick, the neonates will be managed separately with fresh expressed breastmilk [9].

We are presenting a series of representative cases of infants who received breastfeeding from COVID-19 mothers. We’ve described the management of the mothers and babies during breastfeeding, the indication of breastfeeding depending on the severity of symptoms and finally breastfeeding in preterm neonates during NICU admission.

## Methods

This is a retrospective case series study that recruited 23 consecutive mothers with COVID-19 after delivery from 14 March to 14 April 2020. The study was approved by the University Hospital Puerta de Hierro Research Ethics Committee and Informed Consent was obtained from all patients. One patient revoked her Informed Consent with 22 patients remaining in the study.

The objective of the study was to describe the types of lactation of the COVID-19 mothers, to identify if there were added difficulties and to assess the risk of infection of the newborns.

Data was obtained from medical records and by phone survey, including: maternal age, gestational age, timing of maternal infection (prior or after birth), symptoms or pneumonia, type of delivery, medications prior and after birth, neonatal Reverse Transcription Polymerase Chain Reaction (RT-PCR) testing, timely initiation of breastfeeding, skin to skin contact, type of feeding, breastfeeding complications, follow-up, infant infection, and weight gain.

The protocols for COVID-19 treatment at our hospital were: a) Protocol 1: Mainly symptomatic treatment; b) Protocol 2: hydroxychloroquine 400 mg/12 h × 1 day, 200 mg/12 h × 5 days + azithromycin 500 mg × 5 day; c) Protocol 3 for pregnant patients: Protocol 1 × 5–14 days + darunavir (800 mg) + ritonavir (100 mg) daily; and d) Protocol 4 in puerperium: Protocol 1 × 5–14 days + lopinavir (400 mg) + ritonavir (100 mg) daily.

After delivery, all neonates were tested for SARS-CoV 2 in the first two hours of life by quantitative RT-PCR on samples from the respiratory tract (nasopharyngeal swab). During the COVID-19 pandemic, we have promoted the early or timely feeding and skin-to-skin contact in the delivery room. Timely initiation was defined as the one that occurs spontaneously within the first hour after birth. Temporary separation of mothers with COVID-19 was considered when rooming-in did not occur and/or skin-to- skin contact was avoided.

According to the standard WHO definitions, newborn feeding were classified as follows: a) Exclusive Breastfeeding: breastmilk, including milk expressed, donor or from a wet nurse; b) Complementary Feeding: breastmilk, including milk expressed, donor or from a wet nurse and may also receive infant formula; and c) infant formula. All the indicators described above, measure feeding in the previous 24 h [10].

All mothers with COVID-19 at hospital discharge received Lactancia materna ante la pandemia de Coronavirus COVID-19 (IHAN) breastfeeding written recommendations [11]. Follow-up of mothers with COVID-19 infection and newborns was done by phone by a pediatrician during a period of 14 days every three days and then by obstetricians.

## Results

Twenty-three consecutive deliveries by mothers with COVID-19 were attended at our Hospital during the first thirty days of the pandemic and 22 were included in this study. The median age of the mothers was 34 years (range, 19–43) and the median gestational age was 38 + 5 weeks (range, 31 + 6–41 + 3 weeks). All mothers were COVID-19 infected prior to delivery. Demographics of the case series study are shown in Table 1.

**Table 1:** - General features of 22 mothers with COVID-19

Case	GA (wg)	Age (yr)	Maternal infection	Symptoms/ Pneumonia	Medications PB	Type of Delivery	RT-PCR	Medications puerperium	Breastfeeding complications	Infant complications	Infant COVID-19 infection	FU (Mth)
**1**	31 + 6	38	PB	No	No	S	NEG	Symptomatic	No	NICU: Preterm	No	1.5
**2**	34 + 1	34	PB	Pneumonia	H + L/R	CS	NEG	H + L/R	No	NICU: Preterm	No	2.4
**3**	36 + 0	19	PB	Pneumonia	No	CS	NEG	H + L/R + Az	ICU: HELLP	Neonatal Admission	No	1.3
**4**	37 + 2	30	PB	Pneumonia	H + D/C	S	NEG	Symptomatic	No	No	No	2.2
**5**	37 + 6	39	PB	Mild	Symptomatic	S	NEG	Symptomatic	No	No	No	1.4
**6**	38 + 1	37	PB	Mild	Symptomatic	S	NEG	Symptomatic	No	No	No	2.0
**7**	38 + 2	31	PB	No	Symptomatic	I	NEG	Symptomatic	No	No	No	2.1
**8**	38 + 2	23	PB	Pneumonia	H	S	NEG	H	No	No	No	1.8
**9**	38 + 3	35	PB	No	No	I	NEG	Symptomatic	No	No	No	1.8
**10**	38 + 3	37	PB	No	No	CS	NEG	Symptomatic	No	No	No	1.8
**11**	38 + 6	26	PB	Pneumonia	H + Az	CS	NEG	H + Az	No	No	No	1.8
**12**	39 + 1	38	PB	Mild	Symptomatic	S	NEG	Symptomatic	No	No	No	1.4
**13**	39 + 2	31	PB	No	No	S	NEG	Symptomatic	No	No	No	1.8
**14**	39 + 2	31	PB	No	No	S	NEG	Symptomatic	No	No	No	1.7
**15**	39 + 3	43	PB	No	No	I	NEG	Antibiotic	Mastitis	Hospital admission	No	1.8
**16**	39 + 3	39	PB	Mild	Symptomatic	S	NEG	Symptomatic	No	No	No	1.7
**17**	39 + 6	31	PB	No	No	I	NEG	Antibiotic	Mastitis	No	No	1.7
**18**	40 + 0	34	PB	No	No	S	NEG	Symptomatic	No	No	No	1.5
**19**	40 + 3	34	PB	No	No	S	NEG	Symptomatic	No	No	No	1.5
**20**	40 + 4	38	PB	Mild	Symptomatic	S	NEG	Symptomatic	No	No	No	1.8
**21**	41 + 1	37	PB	No	No	S	NEG	Symptomatic	No	No	No	1.5
**22**	41 + 3	29	PB	Mild	Symptomatic	S	NEG	Symptomatic	No	No	No	1.4

**Abbreviations:** GA: gestational age; WG: week gestation; yr: years; PB: COVID-19 prior birth; RT-PCR: Reverse Transcription Polymerase Chain Reaction to infant nasal swab 2 h after birth; AB: after birth; FU: Follow-up; Mth: months; H: Hydroxychloroquine; Az: Azithromycin; L: Lopinavir; D: Darunavir; R: Ritonavir; S: Spontaneous; CS: Cesarean section; I: instrumental; NEG: negative; NICU: neonatal intensive care unit; ICU: intensive care unit

Out of 22 mothers with COVID-19, 11 (50%) were symptomatic. Of all patients, four received treatment for COVID-19 prior to delivery and four in the puerperium. Twenty-two newborns tested negative for SARS-CoV-2 by RT-PCR nasopharyngeal swabs and two born preterm needed admission to the NICU. Clinical course of mothers and newborns are described in Table 1.

Timely initiation and skin to skin contact were performed at delivery room in twelve and thirteen of cases, respectively. Of twenty-two patients, only two patients expressed their desire of lactation suppression. Most newborns (72.7%) were fed with exclusive breast milk, although six of them (37.5%) required transitory complementary feeding until exclusive breastfeeding was achieved. In addition, complementary feeding with infant formula was required in cases 13 and 16, one of them switched to feeding only with infant formula after seven weeks (case 13).

The median length of follow-up was 53.5 days (range, 30–73 days) and the median weight gain was 1351.5 g (range, 270–2300 g). Out of 22 newborns, 81.8% were breastfed at month, and the percentage dropped to 77.3% at censoring date. Throughout follow-up period, all newborns were free of COVID-19 infection. Results are shown in Table 2.

**Table 2:** - Breastfeeding follow-up of 22 mothers with COVID-19

Case	Skin to skin contact in 1st hour	Early initiation of breastfeeding in 1st hour	1st week	2nd week	3rd week	4th week	5th week	6th week	7th week	8th week	9th week	10th week	11th week	WG (gr)
**1**	No	No	**D + Ex**	**D + Ex**	**E**	**E**	**E**	**E**	**E**	NA	NA	NA	NA	1303
**2**	No	No	**D**	**D**	**D + Ex**	**E + F**	**E + F**	**E + F**	**E**	**E**	**E**	**E**	**E**	1945
**3**	No	No	**D**	**D**	**E**	**E**	**E**	**E**	NA	NA	NA	NA	NA	270
**4**	No	No	**F**	**F**	**F**	**F**	**E**	**E**	**E**	**E**	**E**	**E**	NA	2300
**5**	No	No	**D + F**	**F**	**F**	**F**	**F**	**F**	**F**	NA	NA	NA	NA	1655
**6**	No	No	**Ex + F**	**E**	**E**	**E**	**E**	**E**	**E**	**E**	**E**	NA	NA	900
**7**	No	No	**Ex + F**	**E**	**E**	**E**	**E**	**E**	**E**	**E**	**E**	NA	NA	2250
**8**	No	No	**F**	**F**	**F**	**F**	**F**	**F**	**F**	**F**	NA	NA	NA	1430
**9**	Yes	Yes	**E**	**E**	**E**	**E**	**E**	**E**	**E**	**E**	NA	NA	NA	1560
**10**	Yes	Yes	**E**	**E**	**E**	**E**	**E**	**E**	**E**	**E**	NA	NA	NA	1100
**11**	Yes	Yes	**E**	**E**	**F**	**F**	**F**	**F**	**F**	**F**	NA	NA	NA	500
**12**	Yes	No	**F**	**F**	**F**	**F**	**F**	**F**	NA	NA	NA	NA	NA	900
**13**	Yes	Yes	**S**	**S**	**S**	**S**	**S**	**S**	**S**	**F**	NA	NA	NA	1300
**14**	Yes	Yes	**E**	**E**	**E**	**E**	**E**	**E**	**E**	**E**	NA	NA	NA	1200
**15**	No	No	**E**	**E**	**E**	**E**	**E**	**E**	**E**	**E**	NA	NA	NA	2020
**16**	Yes	Yes	**E**	**S**	**S**	**S**	**S**	**S**	**S**	**S**	NA	NA	NA	1700
**17**	Yes	Yes	**E**	**E**	**E**	**E**	**E**	**E**	**E**	**E**	NA	NA	NA	300
**18**	Yes	Yes	**E**	**E**	**E**	**E**	**E**	**E**	**E**	NA	NA	NA	NA	1412
**19**	Yes	Yes	**E**	**E**	**E**	**E**	**E**	**E**	**E**	NA	NA	NA	NA	1038
**20**	Yes	Yes	**D + Ex**	**E**	**E**	**E**	**E**	**E**	**E**	**E**	**E**	NA	NA	2120
**21**	Yes	Yes	**E**	**E**	**E**	**E**	**E**	**E**	**E**	NA	NA	NA	NA	1400
**22**	Yes	Yes	**E**	**E**	**E**	**E**	**E**	**E**	**E**	NA	NA	NA	NA	1300

**Abbreviations:** WG: Weight gain (birth to follow-up); gr: grams; D: Donated Breast Milk; Ex: Express Breast Milk; S: Supplementing with formula; E: Exclusive Breast Milk; F: Formula; NA: Not available

## Case presentations

In situations of admission to special units or when mothers presented symptoms, the cases were managed as follows, in order to achieve exclusive breastfeeding:

### NICU or ICU admission

*Case 1 and 2:* Newborns of two preterm deliveries needed Neonatal Intensive Care Unit (NICU) admission, one because of a respiratory distress syndrome and the other for hemolytic anemia. There were no timely feeding and no skin-to-skin contact at delivery room due to prematurity and transfer to the neonatology unit. After 24 h, oral feeding was done in case 1, with pasteurized donor human milk and expressed breast milk until the 17th day and from then with exclusive breast milk. In case 2, feeding was done with pasteurized donor human milk until the 18th day, followed by a period of three weeks of supplementing breastmilk with infant formula before achieving exclusive breastfeeding.

*Case 3:* Mother required Intensive Care Unit (ICU) admission due to HELLP syndrome and her newborn was transferred to the neonatology unit. Timely feeding and skin to skin contact was not done. The newborn was fed by pasteurized donor human milk during the first 14 h after birth. Subsequently, breastfeeding with expressed breast milk was done up to the 16th day and from then exclusively breastfeeding.

**Symptomatic mothers:** Of 11 symptomatic patients, 5 (22.7%) were due to pneumonia and 6 (27.3%) cases due to mild symptoms.

*Cases 4, 8, and 11* were the remaining mothers with COVID-19 and pneumonia. Case 4 completed treatment prior delivery and case 8 and 11 after delivery. Timely feeding and skin to skin contact was done only in case 11. Case 4 was fed with infant formula until the 4th week and then restarted exclusive breastfeeding. Case 8 underwent lactation suppression from the beginning. In case 11, exclusive breastfeeding occurred until the 2nd week, then infant formula was used since the mother decided to stop breastfeeding.

*Cases 5, 6, 12, 16, 20 and 22:* Timely feeding and skin to skin contact were done in 50 and 66.7% of cases, respectively. Due to mother’s symptoms, cases 5, 6 and 20 required supplements for 48 h (cases 5: pasteurized donor human milk + infant formula; case 20: pasteurized donor human milk + expressed breast milk; and case 6: expressed breast milk +infant formula). Case 5 continued with infant formula due to lactation suppression and case 6 and 20 with exclusive breast milk. Case 16, after one week of exclusive breastfeeding, began supplementing with infant formula based on nutritional requirements. Finally, case 12 underwent lactation suppression due to mother’s desire, while case 22 was fed with exclusive breastfeeding from the beginning.

*Cases 7, 9, 10, 13, 14, 15, 17, 18, 19 and 21* were the asymptomatic mothers with COVID-19 infection. Timely feeding and skin to skin contact were done in 90% of them. All cases have been exclusively breastfed to date, except case 7 who used expressed breast milk and infant formula during the first 48 h due to mother and neonate separation, and case 13 that started exclusive breastfeeding for 4 days, continuing with complementary infant formula feeding until the 7th week and from the 8th week, the baby was infant formula fed.

## Discussion

SARS-CoV-2 virus spreads mainly by droplet transmission, although it has also been detected in blood and stool samples [12–14]. So far, the presence of SARS-CoV 2 in placenta, cord blood, amniotic fluid or breast milk has not been demonstrated [6–8, 15, 16]. Horizontal contagion between mother and newborn through droplets is the one we need to be more vigilant.

Experience regarding other respiratory virus pandemics are based in a largest series of cases about SARS infection includes 12 pregnant patients of which only five infants were born with no evidence of perinatal transmission [17]. Although no information about breastfeed is reported [17]. The Society of Obstetricians and Gynaecologists of Canada recommends SARS infected mothers not to breastfeed until recovered from infection as well as isolation from the neonate until the mother is no longer infectious [18]. Pregnant women affected with MERS infection appears to have worse clinical outcomes that patients with SARS. Only 11 pregnancies associated cases have been documented with 91% having adverse clinical outcomes [19]. There is no experience reported on breastfeeding and MERS infection.

The most important societies, such as WHO, UNICEF, ISUOG, RCOG and ABM promote breastfeeding in this COVID-19 pandemic but with special precautions. They also encourage, whenever possible, the skin-to-skin care, especially after birth in order to facilitate their adaptation to the outside world (stabilizing baby’s temperature, breathing rate, heart rate, and blood sugar) and the establishment of breastfeeding [20–24]. The directions of Italian Society of Neonatology (SIN), Spanish Society of Neonatology (SeNeo) and Union of European Neonatal & Perinatal Societies (UENPS) follow these recommendations and they promote the joint management of the mother and her infant [25]. As well, they warn that it is important to practice respiratory hygiene, including during feeding, such as the use of a medical mask, perform hand hygiene before and after contact with the infant, and routinely clean and disinfect surfaces where the symptomatic mother was in contact with [25, 26].

When maternal general health impedes direct breastfeeding or in cases of separation of mother and neonate, mothers should be encouraged and supported to express milk and safely provide this fresh breastmilk to their infants, while applying appropriate hygiene measures [23]. Additionally, expressed breast milk should not be pasteurized because it reduces the biological and immunological value of human milk [20, 21].

However, the current recommendations in this pandemic from de Center for Disease Control and Prevention (CDC), and also from the Chinese Pediatrics COVID-19 Working Group, should be considered, as the first choice, to separate temporarily the mother who has confirmed COVID-19 or is “mother under investigation”, from the newborn to reduce the risk of transmitting the virus [27]. During this period, they recommend the expression of milk with adequate hygiene measures, in order to maintain lactation once they are in direct contact with their children [27–29]. On the other hand, the other societies claim that the impact on separation of mothers and neonates might result in infant formula feeding [20].

### Breastfeeding advice during the COVID-19 outbreak

According with the new evidence, the Breastfeeding Committee at Puerta de Hierro University Hospital in Madrid (Spain) approved the breastfeeding in COVID-19 newly born with the adequate individual protection measures and with the informed consent of the mother.

Even though there does not seem to be vertical transmission between mother and child, after birth the newborn is susceptible to person-to-person spread by being in contact with his mother. For this reason at the beginning of the pandemic no contact between the newborn and the mother was allowed and breastfeeding was not recommended [30], but soon after international recommendations were established suggesting that the benefits of breastfeeding and the mother and child connection outweighed the risk of transmission [20, 24, 31].

From the emotional wellbeing perspective, avoiding mother-baby separation following delivery also enhances mother-baby bonding process. Breastfeeding and early mother-baby contact may facilitate mother-baby bond [32]. Breastfeeding also decreases the risk of developing postpartum depression [33]. Whenever possible, breastfeeding should be encouraged at any time. For mothers and babies who were separated due to their medical condition, breastfeeding may help in the bonding process as it can protect against mental health problems. In our case series study of mothers with COVID-19, close to 65% of them performed skin to skin contact with their newborns and close to 55% of them achieved spontaneously breastfeeding during the first hour.

There are a number of precautions during breastfeeding that should be followed to minimize the risk of transmission [34]: practicing respiratory hygiene (wearing a face mask or suitable alternative), washing hands thoroughly before and after contact with the baby, routinely cleaning and disinfecting any surfaces touched, cleaning thoroughly any infant feeding equipment (including breast pumps, bottles and teats) before and after use and avoid falling asleep with the baby. In our study we have not diagnosed any infections in newborns.

In this scenario, we identified three turning points which may be left unanswered in the discussion and based on our recent experience, we believe that the best approach to solve these problems are:

a) The first issue is that breastfeeding in COVID-19 patients under pharmacological treatment is only acceptable if the parents are informed about the possible risks. The most extended treatments used to treat the COVID-19 infection such as azithromycin, hydroxychloroquine sulfate, lopinavir-ritonavir, tocilizumab and methylpredinosolone, are excreted in very small amounts in breast milk [35–41]. Although no specific studies have been conducted, it seems that most common medicines used for SARS-CoV-2 infection are safe and compatible with breastfeeding, so there are no contraindications or special recommendations to follow regarding the pharmacological treatment that breastfeeding mothers receive. The parents should be informed about this expected minimal exposure of their newborns to the medications.

Our data suggest the compatibility between breastfeeding and treatment for mothers with COVID-19. Most of them (80%), chose to breastfeed their babies. Additionally, for mothers who are temporarily separated from their babies, a transitory supplementation feeding (with pasteurized donor human milk or infant formula) could be a solution until exclusive breastfeeding can be establish.

b) A second problem is that breastfeeding in COVID-19 patients with severe symptoms or with high viral load could increase viral transmission. When mothers and neonates are separated the recommendations are to express milk to provide it to their infants, but when the mothers are unwell, with fever, in need of oxygen therapy and are even admitted to the intensive care unit, expressing milk might be a real challenge for them.

We think that to achieve this, mothers must have an important external help and guidance: specialists who help them with extraction techniques and emotionally support them. They should also be offered support to be able to make the decision to stop breastfeeding if it becomes a burden. These women should explore the possibility of re-lactation (restarting breastfeeding after a gap), wet nursing (another woman breastfeeding or caring for your child) or using donor human milk. Which approach to use will depend on cultural context, acceptability, and the availability of support services. In our study, out of 11 symptomatic patients, nine mothers required temporary separation mother-baby. In these cases, newborns were transfer to the neonatology unit. During this period, 50% of mothers with mild symptoms required supplementing their newborns for 48 h. At the end of the follow-up period, close to 65% of symptomatic mothers with COVID-19 breastfeed their babies: 6 exclusive breastfeeding and 1 supplementing with infant formula.

c) The third issue is how to manage breastfeeding while the newborn is admitted inside the NICU. In these cases, two special challenges are going to be presented to the COVID-19 mothers; the first that they must be isolated and will not be allowed to visit the neonatal units due to the risk of contagion (NICU or ICU admission rate was between 16 to 18 days). And the second, that even if they express their milk and follow the recommendations of the WHO and IHAN, the professionals responsible for their newborns may not accept it because of the risk that the milk or its containers are contaminated. Again, in this situation, emotional support for these mothers must be always offered. In our experience, achieving exclusive breastfeeding is possible even though the infants were admitted or isolated for prolonged periods; the infants of cases 1, 2 and 3 ended up with exclusive breastfeeding after a feed gap were a combination of pasteurized donor human milk and mother’s expressed breast milk was given to them.

In summary, despite all difficulties, 82% of newborns to mothers with COVID-19 were breastfed for the first month, decreasing to 77% at 1.8 months, being 73% of them exclusive breastfeeding, although more than a third of them required complementary breastfeeding temporarily. This event occurred with minimal maternal and newborn complications, and no neonates were infected during breastfeeding.

## Conclusions

Despite the COVID-19 pandemic, the international organizations continue to promote breastfeeding. Our experience shows that breastfeeding is safe with correct infection and control measures to decrease the risk of contagion by droplets and by contact with the respiratory secretions between mother and infant. When mother-baby child separation occurs, supplementing feeding with pasteurized donor human milk or infant formula may be effective until breastfeeding is resumed.

## Data Availability

Patient records are available.

## Abbreviations

SARS: Severe Acute Respiratory Syndrome
SARS-CoV-2: Severe acute respiratory syndrome coronavirus 2
COVID-19: Coronavirus disease 2019
NICU: Neonatal Intensive Care Unit
MERS: Middle East Respiratory Syndrome
WHO: World Health Organization
UNICEF: United Nations International Children’s Emergency Fund
ISUOG: International Society of Ultrasound in Obstetrics and Gynecology
RCOG: Royal College of Obstetricians and Gynaecologists
ABM: Academy of Breastfeeding Medicine
SIN: Italian Society of Neonatology
SeNeo: Spanish Society of Neonatology
UENPS: Union of European Neonatal & Perinatal Societies
CDC: Disease Control and Prevention
